# Employees and the National Insurance Act

**Published:** 1912-05-25

**Authors:** 


### NATIONAL INSURANCE ACT DEPARTMENT.

### EMPLOYEES AND THE NATIONAL INSURANCE ACT.

### XII.—The Insurance of Hospital Nurses.

If it had been possible to obtain the opinion of trained hospital nurses before the National Insurance Act became law as to its application to themselves, it is probable the result would have been an almost unanimous demand for exemption from insurance. It would probably be equally true to assume that a large majority of the profession even now do not consider it necessary to insure against sickness, and for this reason they are opposed to the National Scheme about to be brought into operation. Cogent reasons may be urged in support of this view. Most hospital nurses have enjoyed in the past, as a matter of fact, if not as a matter of right, the privilege of free medical attendance and treatment in all minor illnesses whilst engaged in their hospital duties. In more protracted illness they have usually been cared for with kindly consideration, even though full remuneration may not have been paid for the whole period of their incapacity. These benefits have been incidental to their calling, and have been provided without the necessity of direct contributions. Seeing, then, that medical treatment and attendance and payments during sickness are the principal benefits provided by the Act, and that weekly contributions must be paid compulsorily in order that they may be obtained, it is little wonder that some resentment should be caused. The National Insurance Act is now, however, the law of the land, and its provisions cannot be modified except by an amending Act, and little hope can be entertained of any special exemption of nurses from its operation even by this means.

#### Reasons for Insurance.

There are weighty reasons against the exclusion of nurses, as we shall show. In the first place, a nurse's employment is not essentially permanent, at least so far as her hospital appointment is concerned. She may, and frequently does leave the hospital, either on marriage, or to take up private practice. Although she may not require the insurance whilst engaged at a hospital there is no guarantee that she may not subsequently require the benefits which the Act provides. Then again, in addition to medical and sickness benefits, there are available maternity and sanatorium benefits. But perhaps more forcible than either of these arguments is the fact that the Act is framed on national lines, and the specific exemption of one profession would have led to similar demands from others, which could not logically have been refused. Nurses in voluntary hospitals, as a body, must therefore conform to the Act's requirements, and a clear understanding of these and of the benefits that may be derived is desirable.

#### Nurses as Employed Contributors.

On July 15 next all nurses in the employ of voluntary hospitals will from that date become employed contributors, unless they are in receipt of a salary which, together with the estimated value of the board and lodging provided, exceeds the rate of £160 per annum, or unless they have independent means, not dependent upon their personal exertions, of the value of at least £26 per annum. This means that the hospital authorities, as their employers, will be responsible for seeing that contributions are paid on their behalf. As it is the normal practice for board and lodging to be provided for the nurses, they do not come, as a class, within the scope of the special provisions for reduced contributions which are applicable when the contributor is paid at a rate of remuneration not exceeding 2s. 6d. a working day. The *ordinary* weekly rate of contribution will be 6d., made up of two equal payments of 3d. each, on behalf of the hospital and the nurse respectively. The former must be borne by the hospital, whilst the latter may be recovered from the nurse by deduction from the wages paid. Nurses in Irish hospitals will not have the advantage, under the Act, of free medical attendance and treatment, and their contributions will accordingly be at the reduced rate of 4½d. a week, of which 2½d. is the hospital's contribution and 2d. is the nurse's share, these amounts being payable and recoverable in the same manner as in Great Britain. These are the *ordinary* rates of contribution, as provided by the Act, but it is very likely that advantage may be taken of a special section (47), which makes provision for the case in which the employer is liable to pay wages during sickness.

#### When Wages are Paid During Illness.

The Insurance Commissioners have power under this section to make special orders specifying any classes of employment in which a custom or practice is shown to their satisfaction to prevail, according to which the persons employed receive full remuneration during periods of disease or disablement. Any employer who comes within such order is required to furnish the Commissioners with a formal notice of his intention to avail himself of the advantages conferred. If no such order has been made, and an employer wishes to avail himself of the benefit that could be obtained if an order had been granted, he may apply to the Insurance Commissioners, and if, after ascertaining the views of the persons employed, they think fit, they have the power to extend the provisions of the section to such applicant. The advantage to be obtained under such an order is a reduction from the ordinary rates of contribution, which, instead of being 7d. a week for men and 6d. a week for women, will be 5d. and 4½d. respectively in Great Britain, and instead of 5½d. for men and 4½d. for women will be 3½d. and 3d. respectively in Ireland. It will only be possible, however, for this advantage to be obtained provided the employer undertakes to pay full remuneration to *every* such employee during any period or periods not exceeding six weeks in the aggregate in any one year during which such person may be suffering from any disease or disablement which commenced while in his employment. It is to be particularly noted that the scheme must apply to the whole staff in the particular form of employment, and not to specified individuals only. If it had been left to the option of the employer to take advantage of the scheme for such of his employees only as he desired, he would have had the option of selecting the most healthy members of the staff and guaranteeing their remuneration, whilst passing over to the approved societies for full rates of benefit those of his employees who enjoy less favourable health. An option of this description would have been most undesirable. It should also be noted that the sickness must commence while in his service, but in the event of the illness extending beyond the term of the employment the continuation of the payment of wages will depend upon the character of the contract of service. When the insured person is paid a weekly or monthly wage, and is subject to a week's or a month's notice, the payment of the full remuneration must be continued for the full aggregate period of six weeks, notwithstanding that this may extend beyond the term of the employment. When, however, there is a contract of service for a period of not less than six months certain the employer must undertake to pay the full remuneration during any period of disease or disablement lasting less than six weeks, and for the first six weeks of any sickness lasting for a longer period, even though in total this may be a larger sum than six weeks' remuneration in a year, but he is not liable to continue the remuneration after the expiration of the term of the contract.

### Larger Benefits and Smaller Contributions.

It will thus be seen that the advantage obtainable under the section of reduced contributions is gained in consideration of the employer undertaking as a definite liability the payment of full wages during illness. Although this is so, the employer does not reap the full benefit of the reduced contributions himself. In all cases the employed contributor will obtain a reduction of 1d. a week in his or her personal contribution, whilst the employer will obtain relief to the extent of 1d. a week for his male employees and ½d. a week for his female employees. This is a most important section of the Act, because if it be adopted by the employer the insured contributors will obtain double advantage: first, by receiving for the first six weeks of sickness a higher rate of pay than would have been possible under the ordinary scale of benefits; and, secondly, the reduction of contributions. Within the past few days the Insurance Commissioners have indicated in the press that they are prepared to consider applications under the section, and in view of the advantages to be derived thereunder those nurses in voluntary hospitals who, in the past, have received full wages whilst suffering from illness, would be well advised to bring the matter to the notice of their superiors, with a view to its consideration by the governors.

### The Choice of an Approved Society.

We have so far spoken quite generally of the contributions, but the question immediately arises—To whom are the contributions to be paid? The choice lies with each individual nurse. No compulsion of any description may be exercised in the matter by the hospital authorities, or by any other person or persons. There are two ways in which the choice may be exercised. The contributions may be paid either to the Post Office—in which case the nurse will become a deposit contributor—or to an approved society, that is a society that has obtained the formal approval of the Insurance Commissioners to its undertaking of the administration of benefits under the Act. The nurse who elects to become a deposit contributor will not really be insured at all. Her contributions will be carried to her personal account, and thereout there will every year be paid in advance the sum required to meet the cost of medical and sanatorium benefits and the cost of their administration. Such sums as remain will be available, so far as they will go, for sickness and disablement allowances, and as soon as the fund is exhausted benefit will cease.

We cannot, therefore, advise any nurse to become a deposit contributor. Her alternative course is to join an approved society, and she will have many such societies from which to choose. At first sight it may perhaps appear to be of no consequence which society is chosen, because they will all administer the same statutory benefits. On closer consideration, however, it becomes apparent that much will ultimately depend upon the initial choice. It must be remembered that the societies must be self-governing, which means that they will be subject to the control of the majority of the members. Later on when there are profits or losses, arising from the working of the Act, to be distributed among the members this matter of control will be very important, and if for no other reason nurses will be well advised to join a society formed with the sole object of benefiting members of their own profession. Such a society is being promoted by the Royal National Pension Fund for Nurses, and is to be known as the Nurses' Insurance Society, and it should command the unanimous support of the profession. It is not too early to commence definite steps towards becoming a member, in fact the earlier they are taken the better it will be, not only in the interests of the society, but in the interests of the nurses themselves. Forms of application for membership, with full information, may be obtained from the Secretary, Nurses' Insurance Society, 15 and 16 Buckingham Street, Strand. London, W.C.

---

**NOTE.—Questions and Correspondence on all doubtful points are invited, and should be addressed to the Insurance Editor, The Hospital, 28 Southampton Street, Strand.**

